# Disruption of the Opal Stop Codon Attenuates Chikungunya Virus-Induced Arthritis and Pathology

**DOI:** 10.1128/mBio.01456-17

**Published:** 2017-11-14

**Authors:** Jennifer E. Jones, Kristin M. Long, Alan C. Whitmore, Wes Sanders, Lance R. Thurlow, Julia A. Brown, Clayton R. Morrison, Heather Vincent, Kayla M. Peck, Christian Browning, Nathaniel Moorman, Jean K. Lim, Mark T. Heise

**Affiliations:** a Department of Microbiology & Immunology, University of North Carolina at Chapel Hill, Chapel Hill, North Carolina, USA; b Department of Genetics, University of North Carolina at Chapel Hill, Chapel Hill, North Carolina, USA; c Department of Microbiology & Molecular Genetics, University of Pittsburgh, Pittsburgh, Pennsylvania, USA; d Department of Microbiology, Icahn School of Medicine at Mount Sinai, New York, New York, USA; e Department of Biology, University of North Carolina at Chapel Hill, Chapel Hill, North Carolina, USA; University of Pittsburgh School of Medicine

**Keywords:** alphavirus, chikungunya, opal stop codon, viral pathogenesis

## Abstract

Chikungunya virus (CHIKV) is a mosquito-borne alphavirus responsible for several significant outbreaks of debilitating acute and chronic arthritis and arthralgia over the past decade. These include a recent outbreak in the Caribbean islands and the Americas that caused more than 1 million cases of viral arthralgia. Despite the major impact of CHIKV on global health, viral determinants that promote CHIKV-induced disease are incompletely understood. Most CHIKV strains contain a conserved opal stop codon at the end of the viral nsP3 gene. However, CHIKV strains that encode an arginine codon in place of the opal stop codon have been described, and deep-sequencing analysis of a CHIKV isolate from the Caribbean identified both arginine and opal variants within this strain. Therefore, we hypothesized that the introduction of the arginine mutation in place of the opal termination codon may influence CHIKV virulence. We tested this by introducing the arginine mutation into a well-characterized infectious clone of a CHIKV strain from Sri Lanka and designated this virus Opal524R. This mutation did not impair viral replication kinetics *in vitro* or *in vivo*. Despite this, the Opal524R virus induced significantly less swelling, inflammation, and damage within the feet and ankles of infected mice. Further, we observed delayed induction of proinflammatory cytokines and chemokines, as well as reduced CD4^+^ T cell and NK cell recruitment compared to those in the parental strain. Therefore, the opal termination codon plays an important role in CHIKV pathogenesis, independently of effects on viral replication.

## IMPORTANCE

Chikungunya virus (CHIKV) is a mosquito-borne alphavirus that causes significant outbreaks of viral arthralgia. Studies with CHIKV and other alphaviruses demonstrated that the opal termination codon within nsP3 is highly conserved. However, some strains of CHIKV and other alphaviruses contain mutations in the opal termination codon. These mutations alter the virulence of related alphaviruses in mammalian and mosquito hosts. Here, we report that a clinical isolate of a CHIKV strain from the recent outbreak in the Caribbean islands contains a mixture of viruses encoding either the opal termination codon or an arginine mutation. Mutating the opal stop codon to an arginine residue attenuates CHIKV-induced disease in a mouse model. Compared to infection with the opal-containing parental virus, infection with the arginine mutant causes limited swelling and inflammation, as well as dampened recruitment of immune mediators of pathology, including CD4^+^ T cells and NK cells. We propose that the opal termination codon plays an essential role in the induction of severe CHIKV disease.

## INTRODUCTION

Chikungunya virus (CHIKV) is a mosquito-borne alphavirus in the *Togaviridae* family that is associated with large-scale outbreaks. Infection with CHIKV leads to a febrile illness characterized by severe acute and chronic arthralgia. In 2005, CHIKV reemerged during an outbreak on La Réunion Island off the coast of Africa. This epidemic affected one-third of the island’s population and resulted in an estimated US $66.5 billion in medical expenses (1). In 2013, locally acquired transmission of CHIKV was reported for the first time in the Western Hemisphere on the island of St. Martin in the Caribbean. This resulted in over 1 million suspected cases and 20,000 confirmed cases of CHIKV infection throughout North, South, and Central America (2). Currently, there are no vaccines or antivirals available to prevent or treat CHIKV infection.

The alphavirus genome is encompassed by a positive-sense single-stranded RNA of approximately 11.7 kb (reviewed in reference 3). The nonstructural proteins (nsPs) are encoded at the 5′ end of the genome and translated as a polyprotein precursor that is posttranslationally processed to produce the mature nsP1-4 proteins. During the initial stages of infection, the major nonstructural protein species consist of the polyprotein precursor nsP123 and nsP4. The latter is rapidly cleaved from the polyprotein and is found solely in its mature state. A conserved opal stop codon is located within the C terminus of the nsP3 gene in several alphaviruses (reviewed in reference 3). Read-through of the opal stop codon during translation occurs infrequently and produces full-length nsP1234. Consequently, stoichiometric concentrations of nsP4 are estimated to be much lower than those of the other nonstructural proteins (4). The polyprotein nsP123 and cleaved nsP4 mediate synthesis of the viral negative-sense RNA, which serves as a template for genomic RNA production. Upon further cleavage into the mature proteins nsP1, nsP2, nsP3, and nsP4, replication switches to replicate positive-sense genomic RNA and transcribe subgenomic RNA. The structural polyprotein is translated from subgenomic RNA and consists of capsid, E1, E2, E3, 6K, and transframe (TF) (5, 6). Capsid is cleaved autocatalytically, and the structural proteins are further processed in the endoplasmic reticulum (ER) (7, 8). Together, these proteins assemble around genomic RNA and form virions at the plasma membrane.

Several studies have identified key viral determinants of alphavirus pathogenesis within untranslated regions (UTRs), nonstructural proteins, and structural proteins (9–12). Mutations within the viral nonstructural proteins and envelope glycoproteins modulate type I interferon (13–16), host cell shutoff (17), and the stress response (18, 19). More recently, other work has explored the sequence diversity within clinical isolates of CHIKV from the Caribbean outbreak. Through this analysis, a novel 3′ UTR sequence that allowed for enhanced replication in mosquito cells was discovered (20). These data corroborate studies of both CHIKV and other alphaviruses that indicate that mutations in the UTRs can have profound impacts on replication in mosquito vectors (11, 12). However, studies on CHIKV-specific determinants of disease have been fairly limited and have focused largely on host determinants that influence development of severe disease (21, 22). Of those viral determinants that have been identified, a mutation in the viral envelope (E1-A226V) played a major role in permitting CHIKV replication within a novel mosquito vector, which allowed for an unprecedented expansion of CHIKV into naive human populations (10). All together, these data provide a strong rationale for identifying viral determinants of severe CHIKV disease.

Like many alphaviruses, the majority of CHIKV strains encode an opal termination codon at the 3′ end of nsP3 (20, 23, 24). However, some CHIKV isolates encode an arginine residue in place of the opal termination codon, such as SGP007, a CHIKV isolate from a patient in Singapore (25). Studies with Sindbis virus (SINV) have shown that the opal stop codon regulates both the amount of nsP4 produced during infection (4) and posttranslational cleavage of nsP34 (26). During translation of the nonstructural polyprotein during the first few hours after infection, full-length nsP1234 comprises only 5 to 20% of the polyprotein products translated, while nsP123 is produced to much greater abundance (4). Further, introduction of the arginine mutation into SINV results in accumulation of uncleaved nsP34 instead of mature nsP3 (26). O’nyong-nyong virus (ONN), a closely related alphavirus, encodes either an opal termination codon or an arginine codon (27, 28). One study demonstrated that the opal termination codon confers a fitness advantage for ONN in *Anopheles gambiae*, its mosquito host (28). However, infection with a SINV strain that naturally encodes a cysteine residue in place of the opal stop codon leads to severe morbidity and mortality in mice (9). Together, these data suggest that the presence of an amino acid residue in place of the opal stop codon may alter CHIKV pathogenesis.

In this study, we examined a patient isolate of the Caribbean strain of CHIKV for novel viral variants that could impact pathogenesis. We found that this strain of CHIKV, which was isolated in St. Martin, encodes both the conserved opal termination codon and the arginine mutation. Therefore, we tested how mutations in the opal stop codon impact CHIKV replication and disease. To this end, we introduced the arginine mutation into the infectious clone of a Sri Lankan strain of CHIKV that naturally encodes the opal stop codon (29). The resulting virus was designated Opal524R. We found that the presence of the arginine mutation did not significantly impact viral replication in cultured cells or in target tissues *in vivo*, which is in contrast to prior findings with SINV (26). Strikingly, the Opal524R mutant caused less-severe disease in a mouse model than the wild-type virus, as measured by decreased swelling and histopathological damage in the footpads and ankle joints of infected animals. The Opal524R mutant exhibited delayed induction of proinflammatory cytokine and chemokine production and a corresponding reduction of inflammatory leukocyte recruitment into the feet and ankle joints. Therefore, these data indicate that the presence of an opal termination codon within nsP3 has a major impact on CHIKV-induced disease severity.

## RESULTS

### Identification of the Opal524R mutation in the Caribbean strain of CHIKV.

We performed deep sequencing of a clinical isolate derived from a human patient from the island of St. Martin obtained during the CHIKV outbreak in 2013 (GenBank accession no. MG208125). Through this analysis, we identified a thymine-to-cytosine single nucleotide polymorphism (SNP) within the opal termination codon resulting in a coding change to an arginine residue (data not shown). This SNP was found in nearly 70% of reads, making the arginine codon the dominant variant in this isolate. To examine the role of the opal stop codon in CHIKV pathogenesis, we introduced an arginine codon in place of the opal termination codon in an infectious clone of a Sri Lankan CHIKV strain that is associated with severe disease in mouse models of CHIKV-induced arthritis (29). The resulting mutant virus, which was designated Opal524R, was then characterized to test whether replacement of the opal termination codon had any impact on viral replication or the pathogenesis of CHIKV-induced arthritis.

### The Opal524R mutation does not adversely affect CHIKV replication.

**FIG 1:**
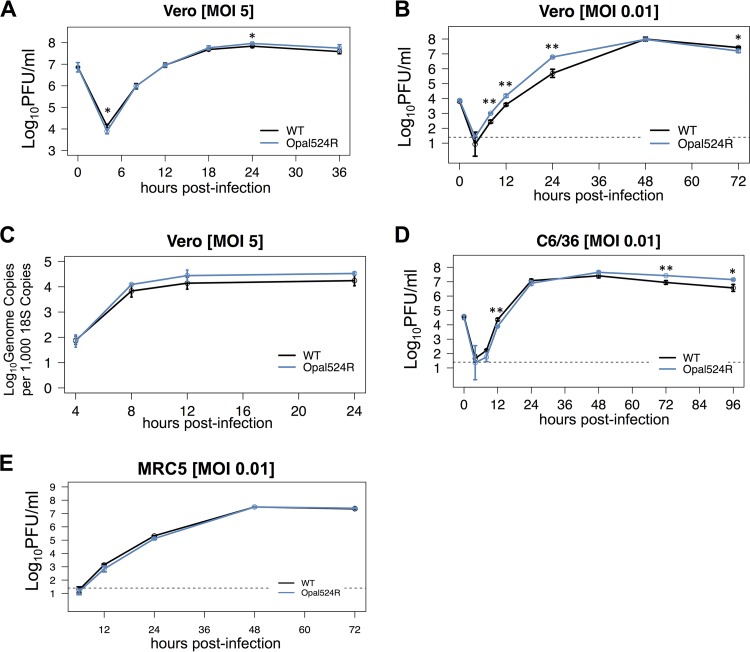
Mutation of the opal stop codon to an arginine does not impair CHIKV replication *in vitro*. (A, B) Vero cells were infected in triplicate at a multiplicity of infection (MOI) of 5 (A) or 0.01 (B) with wild-type CHIKV (WT) or the Opal524R mutant. The supernatant was collected from infected cells at the indicated times postinfection, and its titer was determined by plaque assay on Vero cells. (C) Vero cells were infected in triplicate at an MOI of 5 as described for panel A. At the indicated times postinfection, RNA was extracted for quantitative real-time RT-PCR. (D, E) C6/36 cells (D) or MRC5 cells (E) were infected in triplicate at an MOI of 0.01 with wild-type CHIKV or the Opal524R mutant. The supernatant was collected and its titer was determined as described for panels A and B. Dashed lines represent the limit of detection for the assay. Data were analyzed by ANOVA for significance, with corrections for multiple comparisons. All data are representative of two independent experiments. Statistical significance is indicated as follows: *, *P* < 0.05; **, *P* < 0.01.

Mutations in the opal termination codon and nearby sequence adversely impact multiple aspects of replication in some SINV and CHIKV strains (4, 25, 26, 30). Therefore, we initially tested whether the Opal524R mutation had any impact on viral replication *in vitro*. We found no defects in Opal524R growth at high or low multiplicities of infection (MOI) in Vero cells (Fig. 1A and B) or at a low MOI in *Aedes albopictus* C6/36 cells (Fig. 1D). In fact, the Opal524R mutant showed enhanced replication compared to that of the wild-type virus at early times postinfection in multistep growth curves (Fig. 1B), which is consistent with prior findings for ONN (28). Furthermore, we found that the Opal524R mutant produces genomic RNA with kinetics and abundances similar to those of the parental virus (Fig. 1C). However, Vero cells exhibit impaired interferon signaling. Therefore, we further tested the Opal524R mutant for replication defects in a human cell line with intact innate immunity. When MRC-5 cells, which have a functional type I IFN response, were infected at an MOI of 0.01, we found no defects in replication at early or late times postinfection (Fig. 1E). These results indicate that the presence of the arginine residue in place of the opal termination codon does not overtly impair CHIKV replication *in vitro*.

### The Opal524R mutation alters nsP4 processing.

**FIG 2:**
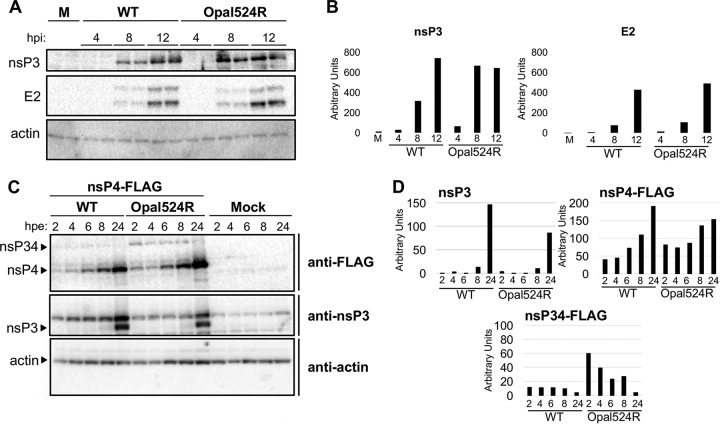
The Opal524R mutation alters levels of nsP34 and mature nsP4 within infected cells. (A, B) Vero cells were infected in duplicate at a multiplicity of infection (MOI) of 5 with wild-type CHIKV or the Opal524R mutant. (A) At the indicated times postinfection (hours postinfection [hpi]), protein was extracted for Western blotting. Blots were probed for the indicated markers. M, mock. (B) Densitometry was performed for nsP3 and E2 using ImageJ software. Band intensity was normalized against that of actin, and the averages of intensities in duplicate lanes from a representative blot are shown. Data are representative of two independent experiments. (C, D) CHIKV replicons containing either the wild-type sequence or the Opal524R mutation were generated. Each replicon contains a C-terminal 3× FLAG tag on nsP4 (nsP4-FLAG). BHK-21 cells were electroporated with 10 μg of Sp6-transcribed RNA of the indicated replicon or mock electroporated in 1× PBS. (C) At the indicated times postelectroporation (hours postelectroporation [hpe]), protein was extracted for Western blotting. (D) Densitometry was performed for nsP3, nsP4-FLAG, and nsP34-FLAG using ImageJ software. Band intensity was normalized against that of actin. Data are representative of two independent experiments.

Previous reports indicated that mutation of the opal stop codon to an amino acid in SINV can impact viral protein production or processing (26, 30). Therefore, we measured levels of nsP3 and E2 in Vero cells infected with either the wild-type virus or the Opal524R mutant. Measurable levels of mature nsP3 were observed beginning at 8 h postinfection for both viruses, although levels were elevated for the Opal524R mutant compared to those of the wild-type virus at this time point (Fig. 2A and B). However, the monoclonal antibody used in these assays appears to be unable to detect nsP3 in the context of nsP123, nsP1234, or nsP34, so we were unable to assess the efficiency of proteolytic cleavage of nsP3 from the nonstructural polyprotein. Both the mature and immature isoforms of E2 were detectable at comparable levels for the two viruses, beginning at 8 h postinfection. We next tested whether nsP4 production or processing was altered by the Opal524R mutation. To measure different isoforms of nsP4, we generated CHIKV replicons with C-terminal FLAG tags after nsP4 for both the wild-type and Opal524R viruses. BHK-21 cells were electroporated with RNA from either replicon, and nsP4 production was measured using antibodies against FLAG. The Opal524R mutant produced higher levels of mature nsP4-FLAG beginning at 2 h postinfection and continuing through 8 h postinfection, suggesting that the Opal524R mutation enhances translational read-through of the nsP4 open reading frame (Fig. 2C and D). In addition, levels of immature nsP34-FLAG were also higher for the Opal524R virus at all time points measured (Fig. 2C). Overall, these results suggest that enhanced translational read-through by the Opal524R mutant results in increased levels of both the nsP34-FLAG precursor and mature nsP4-FLAG within infected cells.

### The Opal524R mutant fails to induce severe disease in a mouse model.

**FIG 3:**
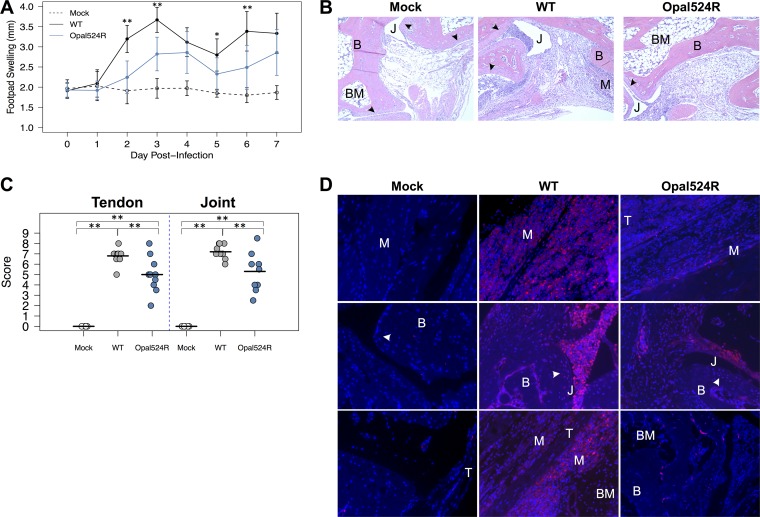
The opal stop codon is required by CHIKV to induce severe disease. (A) Twenty-four-day-old C57BL/6J mice were either mock infected (*n =* 6) or infected with 100 PFU of wild-type CHIKV or the Opal524R mutant (9 to 10 mice per group) in the left rear footpad. Mice were monitored for 7 days, and footpad swelling was measured in millimeters using calipers. (B, D) Mice from panel A were sacrificed at day 7 postinfection and perfused intracardially with paraformaldehyde. Tissue sections were prepared from the ipsilateral foot and embedded in paraffin, and 5-μm sections were prepared. Tissues were deparaffinized and stained with either hematoxylin and eosin (B) or nitrotyrosine (D). Representative images of the ankles at a 10× magnification are shown. (C) Hematoxylin-eosin sections from panel B were scored blind for damage to the tendon and ankle joint. Horizontal lines indicate the arithmetic mean. B, bone; BM, bone marrow; J, joint space; M, muscle; T, tendon; arrowhead, synovial lining. Data shown are from at least two independent experiments. Data were analyzed by ANOVA for significance, with corrections for multiple comparisons. Statistical significance is indicated as follows: *, *P* < 0.05, and **, *P* < 0.01.

We next tested whether the Opal524R mutant exhibited altered virulence *in vivo* using a mouse model of CHIKV-induced disease. In this model, footpad infection with wild-type CHIKV results in biphasic footpad swelling and tenosynovitis within the inoculated foot (29). The Opal524R mutant induced footpad swelling over that in mock-infected animals; however, the level of swelling was significantly reduced compared to that caused by the wild-type virus at both early and late times postinfection (Fig. 3A). This suggests that the Opal524R mutant is less virulent *in vivo* than the parental virus in a mouse model of disease.

### Damage and inflammation is reduced in the ankle joints of Opal524R-infected mice.

To further analyze the impact of the arginine mutation on CHIKV-induced disease, mock-infected or CHIKV-infected mice were sacrificed at 7 days postinfection and inflammatory pathology was assessed within the feet and ankle joints on hematoxylin-and-eosin-stained sections. We observed reduced inflammatory cell infiltration within the joints of mice infected with the Opal524R virus, consistent with the attenuation in virus-induced swelling (Fig. 3B). Blind histological analysis also indicated that infection with the Opal524R mutant resulted in less damage within ankle joints and tendons (Fig. 3C). To more closely assess inflammation within joint tissues, we stained for nitrotyrosine accumulation within the ankle joints at day 7 postinfection (Fig. 3D). Under conditions of oxidative stress, superoxide and nitric oxide react to form the free radical peroxynitrite, which causes nitration of tyrosine residues (31, 32). Nitrotyrosine accumulation is a stable indicator of oxidative stress associated with several inflammatory disease states, including celiac disease, rheumatoid arthritis, and CHIKV infection (31–35). We previously found that wild-type CHIKV infection induces oxidative stress within infected feet, as indicated by a pronounced accumulation of nitrotyrosine within these tissues (36). Consistently with these earlier results, infection with wild-type CHIKV resulted in high levels of nitrotyrosine staining in muscle, tendon, and the synovial lining of the joints (Fig. 3D). However, we observed a marked decrease in nitrotyrosine accumulation in all of these regions of the feet and ankle joints of mice infected with the Opal524R mutant compared to that found in mice infected with the parental virus. Together, these data support the conclusion that the opal termination codon contributes to CHIKV-induced disease *in vivo*.

### The Opal524R mutant replicates efficiently *in vivo*.

**FIG 4:**
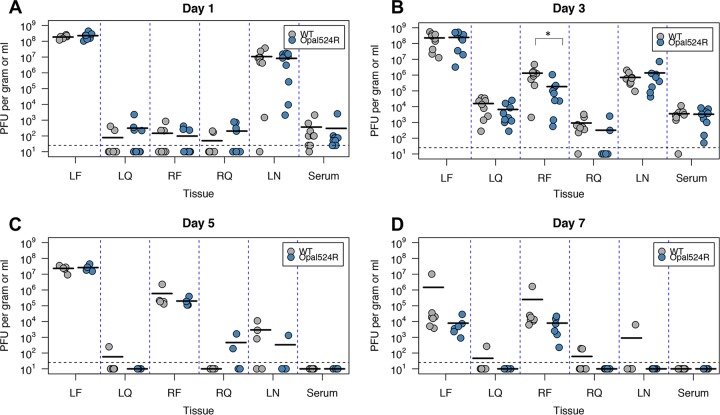
CHIKV replication at target sites *in vivo* is not impaired by mutagenesis of the opal stop codon. Twenty-four-day-old C57BL/6J mice were infected in the left rear footpad with 100 PFU of wild-type CHIKV or the Opal524R mutant (9 to 10 mice per group per time point). Mice were sacrificed at 1 (A), 3 (B), 5 (C), or 7 (D) days postinfection, and the indicated tissues were harvested. Tissues were homogenized and titers were determined by plaque assay on Vero cells. Horizontal bars represent the arithmetic mean. Dashed horizontal lines represent the limit of detection for the assay. LF, left (ipsilateral) foot; LQ, left quadriceps muscle; RF, right (contralateral) foot; RQ, right quadriceps muscle; LN, popliteal lymph node. Combined data from two independent experiments are shown. Data were analyzed by one-way ANOVA for significance, with corrections for multiple comparisons. Statistical significance is indicated as follows: *, *P* < 0.05.

Although the Opal524R mutant replicated efficiently *in vitro*, one likely explanation for the differences in disease induction between these two viruses might be a general defect in viral replication *in vivo*. To address this, we measured replication within both proximal and distal tissues at several times postinfection (Fig. 4). We found that the Opal524R mutant replicates efficiently in all target tissues, including the ipsilateral and contralateral foot and quadriceps muscles and popliteal lymph nodes. Levels of viremia in serum were also equivalent between the two viruses. Therefore, while viral replication was transiently reduced in the contralateral foot at day 3 postinfection (Fig. 4B), these results suggest that the differences in virus-induced swelling and pathology in the inoculated foot cannot be explained by differential *in vivo* replication. It is also possible that the Opal524R virus may revert back to wild type *in vivo*, thereby leading to equivalent levels of viral replication. To test this, subsets of mice (5 mice infected with the wild type; 7 mice infected with Opal524R) were sacrificed at 24 h postinfection and RNA was extracted from the inoculated foot. Sequencing across the nsP3 gene confirmed that the Opal524R mutation is stable and did not revert at 24 h postinfection *in vivo*.

### Immune cell recruitment to the site of infection is limited during Opal524R infection.

**FIG 5:**
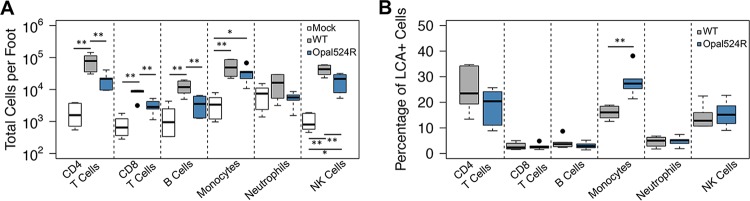
Failure of the CHIKV Opal524R mutant to induce severe disease is associated with low immune cell infiltration. (A, B) C57BL/6J mice were infected at 24 days old in the left rear footpad with 100 PFU of wild-type CHIKV or the Opal524R mutant or mock infected with diluent (6 per group). At 7 days postinfection, mice were sacrificed and perfused intracardially with 1× PBS. The ipsilateral foot was harvested and processed for analysis by flow cytometry. Recruitment of the indicated inflammatory leukocyte populations was measured and expressed as either total cell counts per foot (A) or percentage of LCA^+^ cells (B). Data are representative of two independent experiments. Individual data points, which are represented by a solid dot, represent outliers. These are defined as values that exceed the third quartile plus 1.5 times the interquartile range (IQR) or fall below the first quartile minus 1.5 times the IQR. Statistical significance is indicated as follows: *, *P* < 0.05, and **, *P* < 0.01.

Specific immune cell populations, including monocytes, CD4^+^ T cells, and NK (natural killer) cells, infiltrate the site of CHIKV infection *in vivo* and mediate disease (37, 38). Therefore, the differences in pathology that we observed might reflect differences in levels of recruitment of specific immune cell mediators into the infected joints. We tested this by measuring immune cell recruitment to the site of infection by flow cytometry at day 7 postinfection. The prevalences of several types of immune cells differed when mice were infected with the Opal524R mutant. Recruitment of CD4^+^ T cells and NK cells, two cell populations that contribute to CHIKV-induced inflammatory pathology (37, 38), were reduced in Opal524R-infected mice (Fig. 5A). Overall numbers of CD8^+^ T cells and B cells were also reduced following Opal524R infection, despite the fact that these cell populations have not been implicated as drivers of CHIKV-induced inflammation (Fig. 5A). Interestingly, while monocytes have also been implicated in driving CHIKV pathogenesis (38), we did not observe statistically significant differences in levels of recruitment of monocytes between the Opal524R mutant (based on overall cell numbers) and the wild type. In fact, we found that monocytes comprise a greater percentage of total infiltrating leukocytes (defined as leukocyte common antigen-positive [LCA^+^] cells) during infection with the Opal524R virus than during infection with the wild type (Fig. 5B). Overall, these data suggest that attenuation of swelling during Opal524R infection may be due in part to a failure to recruit high levels of CD4^+^ T cells and NK cells but cannot be explained by differences in monocyte recruitment.

### Proinflammatory cytokines and chemokines are produced to lower levels at day 1 postinfection.

**FIG 6:**
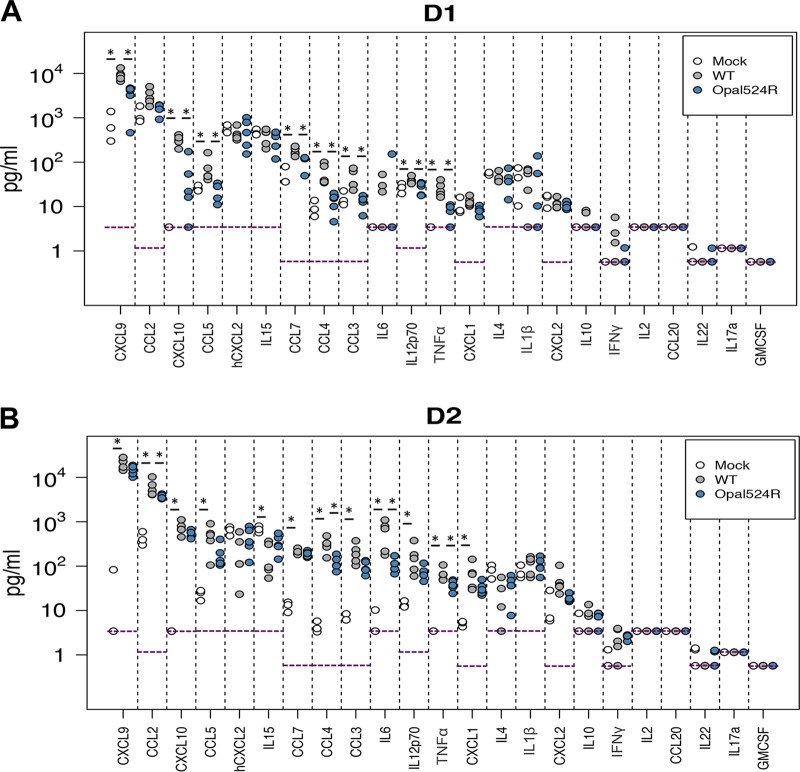
The opal termination codon enhances CHIKV-mediated induction of proinflammatory cytokine and chemokine production. (A, B) C57BL/6J mice were infected at 24 days old in the left rear footpad with 100 PFU of wild-type CHIKV (*n =* 5) or the Opal524R mutant (*n =* 5) or mock infected with diluent (*n =* 3). At days 1 (A) and 2 (B) postinfection, mice were sacrificed and perfused intracardially with 1× PBS, and the ipsilateral foot was harvested and homogenized in PBS. Cytokine production was measured in tissue homogenates by multiplex ELISA. Data were analyzed by the Mann-Whitney *U* test. Asterisks indicate significance after multiple comparisons were corrected with the Benjamini-Hochberg method, using a false-discovery rate of 0.1.

Persistent arthralgia in CHIKV-infected patients is characterized by high levels of certain proinflammatory cytokines, such as interleukin 6 (IL-6), MIG (monokine induced by interferon gamma), and IP-10 (interferon gamma-induced protein 10) (39, 40). Based on the differences described in inflammatory cell recruitment, we hypothesized that differential chemokine activation during Opal524R infection accounts for the lower numbers of CD4^+^ T cells and NK cells recruited to the site of infection. To address this question, we measured cytokine production within the ipsilateral foot early during infection. Infection with wild-type CHIKV induced production of several cytokines at day 1 postinfection, including the proinflammatory cytokines tumor necrosis factor alpha (TNF-α), IL12p70, and the chemokines CXCL9 (CXC-motif chemokine 9) (MIG), CXCL10 (IP-10), CCL3 (CC-motif chemokine 3) (MIP-1α), CCL4 (MIP-1β), CCL5 (RANTES), and CCL7 (MCP3) (Fig. 6A). Protein levels for all of these cytokines were significantly reduced during Opal524R infection. Of these, CXCL9 (MIG), CXCL10 (IP-10), and CCL4 (MIP-1β) are known T cell and NK cell chemoattractants. Further, although we did not observe significant differences in monocyte recruitment to the footpad, CCL3, CCL4, CCL7, and CXCL10 are all known monocyte chemoattractants that were also significantly reduced during Opal524R infection. This difference in early chemokine production is consistent with our findings that CD4^+^ T cells, CD8^+^ T cells, and NK cells are less efficiently recruited during Opal524R infection.

### Levels of cytokine production are comparable between the wild type and Opal524R at day 2 postinfection.

At day 2 postinfection, many of the cytokines induced by wild-type infection were induced to comparable levels by Opal524R infection, with two exceptions. TNF-α and CCL4 (MIP-1β) continued to be produced to significantly lower levels during Opal524R infection (Fig. 6B). Wild-type infection also induced IL-6, CCL2 (MCP-1), and CXCL1 at day 2 postinfection and suppressed IL-15 production compared to levels in mock-infected animals. By comparison, CCL2 and IL-6 were significantly reduced during Opal524R infection at day 2 postinfection. Together, these data suggest that there is a delay in activation of many proinflammatory cytokines early during Opal524R infection. Notably, TNF-α and CCL4 levels were significantly lower during Opal524R infection at days 1 and 2 postinfection (Fig. 6). These differences may account for the reduction in inflammatory cell infiltration and pathology observed in the joint during infection with the Opal524R mutant.

### Cytokine induction is also diminished at the transcript level.

**FIG 7:**
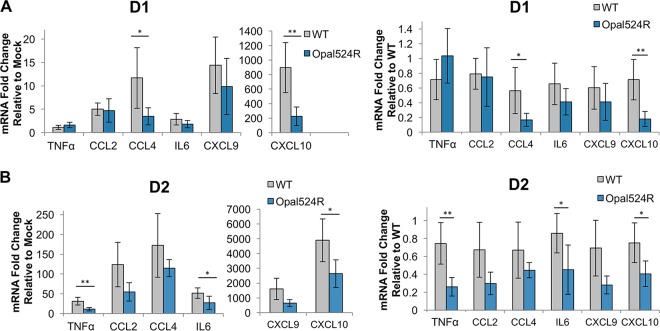
Cytokine gene expression is reduced during CHIKV Opal524R infection. (A, B) Twenty-four-day-old C57BL6/J mice were infected in the left rear footpad with 100 PFU of wild-type CHIKV (*n =* 5) or Opal524R (*n =* 5) or mock infected with diluent (*n =* 3). At days 1 (A) and 2 (B) postinfection, mice were sacrificed and perfused intracardially with 1× PBS, and the ipsilateral foot was harvested in TRIzol. RNA was extracted, cDNA was generated, and cytokine gene expression was assessed by quantitative real-time PCR. Each cytokine was normalized to host 18S gene expression. Fold changes in gene expression relative to mock infection (left) and wild-type infection (right) are shown. Data are representative of two independent experiments. Statistical significance is indicated as follows: *, *P* < 0.05, and **, *P* < 0.01.

We further confirmed differences in cytokine production by measuring mRNA transcript levels for a subset of cytokines by quantitative real-time reverse transcription PCR (qRT-PCR). In mice infected with wild-type virus, transcripts were only modestly elevated at day 1 postinfection for CCL2, CCL4, IL-6, and CXCL9, while CXCL10 transcripts were very highly elevated relative to levels in mock-infected mice (Fig. 7A). Consistently with the reduction in protein levels observed, CCL4 and CXCL10 transcripts were significantly reduced in Opal524R-infected mice relative to those in wild-type-infected mice. TNF-α mRNA levels were not significantly different from those in mock-infected mice for either virus. At day 2 postinfection, both TNF-α and IL-6 transcripts were moderately induced by wild-type virus, while CCL2, CCL4, CXCL9, and CXCL10 mRNA levels were all highly elevated by wild-type virus infection (Fig. 7B). There was a significant reduction in TNF-α, IL-6, and CXCL10 transcripts during Opal524R infection at this time point. These data support the conclusion that the Opal524R mutant differentially induces proinflammatory cytokines and chemokines.

## DISCUSSION

There is presently no consensus on the requirement for the opal termination codon in alphavirus replication. The AR86 strain of SINV and the SFV4 and prototype strains of Semliki Forest virus (SFV) tolerate coding changes in place of the opal termination codon (9, 41), and these mutations can enhance virulence. Furthermore, ONN replication *in vitro* is actually enhanced by introduction of an arginine codon in place of the opal stop codon (28). Conversely, mutations to the opal termination codon and nearby sequence adversely impact multiple aspects of replication in other SINV strains (4, 26, 30). Specifically, translational read-through of the opal codon, proteolytic cleavage of nsP34, production of genomic RNA, and production of infectious particles *in vitro* are all negatively impacted when the opal termination codon or the cytosine base immediately downstream are mutated. Similarly, another study found that nonisogenic CHIKV strains encoding the opal stop codon replicate more efficiently *in vitro* than arginine-containing strains (25).

In this study, we identified a mutation of the opal termination codon to an arginine residue in a patient-derived isolate of CHIKV from the recent Caribbean outbreak, isolated on St. Martin. One other CHIKV sequence available from a patient on St. Martin also contains the Opal524R mutation (GenBank accession number KX262991). However, sequences from patient isolates from other regions of the Caribbean outbreak contain the opal stop codon. Specifically, 10 isolates analyzed from Haiti (GenBank accession numbers KX702401.1, KX702402, and KY415978 to KY415985) and 15 isolates analyzed from Puerto Rico (GenBank accession numbers MF001505 to MF001519) all contain the opal stop codon (42). This may suggest that early isolates of CHIKV from the Caribbean outbreak contained the Opal524R mutation but that this was lost over time. Further sequence analysis of more patient samples from different stages of the outbreak will be required to ascertain whether this occurred.

Earlier work demonstrated that the Opal524R mutation can be tolerated in isolates of CHIKV from other regions (e.g., SGP007, a patient isolate from Singapore) but that the SGP007 isolate exhibited reduced replication efficiency relative to those of other CHIKV isolates (SGP011, another patient isolate from Singapore, and IMT, a La Réunion strain) (25). Our study instead demonstrates that the Opal524R mutation can be efficiently tolerated in the context of CHIKV with no cost to overall replication efficiency. However, there are important differences between these studies that might account for these discrepancies. Our study utilized virus stocks derived from infectious clones, so only the effect of the Opal524R mutation was considered. In contrast, these earlier comparative analyses were based on patient isolates. In particular, the SGP007 strain contains six additional coding changes from the sequences of IMT and SGP011 that may interact with the Opal524R mutation to contribute to the observed replication defects. Together, these reports may suggest that additional linked mutations are required for optimal replication efficiency. Further investigation of such potential linked mutations is under way.

This study demonstrates that the conserved opal termination codon within CHIKV is required for induction of severe disease in a mouse model of CHIKV-induced arthritis. Mutations to the opal stop codon enhance virulence in the AR86 strain of SINV (9) and in the SFV4 strain of Semliki Forest virus (41), but the data reported herein instead suggest that the arginine mutation found in CHIKV does not increase disease severity or replication in a mouse model of disease. These results illustrate that mutations to the opal stop codon may have contrasting effects on the virulences of different alphavirus family members, while also raising the possibility that such mutations may impact other aspects of the CHIKV replication cycle. Furthermore, understanding how the opal termination codon modulates alphavirus virulence and the mechanisms underlying this effect may have important implications for the development of live attenuated vaccines against viruses such as CHIKV. As such, additional studies of the molecular mechanism by which the opal stop codon contributes to CHIKV virulence are warranted.

In this study, we demonstrate that the opal termination codon plays an important role in CHIKV pathogenesis. We observed striking differences between the Opal524R mutant and the parental virus in both immune pathology and tissue inflammation. However, these effects occur independently of any measurable effect on viral replication. Our results do suggest that the Opal524R mutation alters translational read-through of nsP4, resulting in increased expression of both mature nsP4 and the nsP34 cleavage intermediate within infected cells. Further studies are necessary to discern whether these differences in nonstructural protein processing can account for differential disease outcomes. For example, although we did not observe differences in genomic RNA expression in infected cells (Fig. 1C) or altered viral replication in IFN-competent cells (Fig. 1E) or *in vivo* (Fig. 4), we cannot rule out the possibility that increased levels of nsP4 expression alter viral RNA synthesis. These differences may enhance innate immune sensing or cellular stress responses, thereby boosting the early immune response and limiting pathological downstream immune responses. Additional studies to test these possibilities are underway.

While we did not see any negative impact of the Opal524R mutation on replication in an *Aedes albopictus* cell line, C6/36, we cannot rule out the possibility that this mutation will impact other aspects of the viral replication cycle in mosquito vectors. As noted above, studies with the closely related alphavirus ONN have identified the presence of both opal and arginine codons in nsP3 (27, 28). ONN strains carrying the arginine codon exhibited a growth advantage *in vitro*, which is similar to our findings with CHIKV (Fig. 1). However, these studies demonstrated that the opal termination codon enhanced infection in the mosquito vector, which may explain why the vast majority of CHIKV isolates sequenced to date encode the opal termination codon, rather than arginine, at nsP3 position 524. Therefore, additional studies are clearly needed to directly test the potential role of the opal termination codon in CHIKV replication in mosquito vectors.

Currently, there are no effective vaccines available to protect against CHIKV-induced disease. In this study, we show that mutation of the opal stop codon produces a viable mutant that fails to induce severe disease. CHIKV infection in human patients is characterized by induction of several proinflammatory cytokine and chemokine markers, including gamma interferon (IFN-γ), TNF-α, IP-10, and IL-6 (39, 40, 43). Our group and others have demonstrated that similarly high production of these and other cytokines occur very early during CHIKV infection in a mouse model (38–40). These cytokines are induced more slowly during infection with the Opal524R mutant. Therefore, inclusion of opal stop codon mutations within live attenuated CHIKV vaccines containing other attenuating mutations may represent a reasonable strategy to generate a protective immune response. However, additional studies are needed to assess both the safety and the stability of such live attenuated viruses.

In addition to their potential for application in vaccine design, our results may have implications for the development of CHIKV therapeutics. A recent study by Ashbrook et al. found that inhibitors of sodium-potassium ATPases antagonize CHIKV replication and that drug-resistant variants often contain mutations in the opal termination codon (44). Drug-resistant variants containing the Opal524R mutation were also obtained when CHIKV was instead passaged in the presence of difluoromethylornithine (DFMO), a drug that inhibits polyamine biosynthesis and thereby restricts viral replication (45, 46). Interestingly, the Opal524R mutant did not replicate as well as the parental strain in either *Aedes albopictus* mosquitos or zebrafish, but when combined with additional drug-resistant mutations, the Opal524R mutation enhances replication *in vivo* (46). Although these drug-resistant variants have not, to our knowledge, been tested for virulence in a mouse model, our results suggest that these viruses would cause less tissue damage than the parental virus. Therefore, antiviral therapies that result in coding changes to the CHIKV opal stop codon may have the added benefit of selection for less virulent variants.

In summary, our results demonstrate that the opal termination codon plays an essential role in promoting the development of CHIKV-induced arthritis independently of effects on viral replication. Most CHIKV isolates sequenced to date contain opal termination codons at nsP3 position 524; however, we observed no negative impact of the arginine mutation on viral replication. Taken together, these results suggest that the opal termination codon plays an essential role in regulating multiple aspects of the CHIKV replication cycle while also promoting CHIKV-induced inflammatory pathology.

## MATERIALS AND METHODS

### Viruses and cells.

All infectious work with CHIKV was performed under biological safety level 3 conditions using approved standard operating procedures. The clinical isolate of the Caribbean strain of CHIKV used in this study (GenBank accession no. MG208125) was provided by Michael Diamond (Washington University) and was amplified three times in Vero cells prior to receipt by our lab. This isolate was originally obtained from a human patient on the island of St. Martin during the 2013 outbreak and acquired from the World Reference Center for Emerging Viruses and Arboviruses (University of Texas Medical Branch). Virus stocks were amplified in the *Aedes albopictus* C6/36 cell line. The parental strain of CHIKV used in this study was generated from the clinical isolate SL15649, which originated in Sri Lanka (29). The infectious clone pMH56.2 encodes the full-length genome of this isolate and has been previously described (36). An infectious clone for the mutant virus Opal524R was generated through site-directed mutagenesis. Specifically, primer sets encoding the Opal524R mutation (forward, 5′GAGAAGCCCGGGACGCAGAAAAAG; reverse, 5′GACCTGGACCGGTGTCCGACGAGAATATATACCCACCTGCCCTGTCTAGTCGTAACTC) were used. This amplicon was ligated into the pMH56.2 plasmid backbone. The entire ligated region of the purified, clonal plasmid was sequenced to confirm that no spurious mutations were introduced. Virus stocks from both the wild-type and Opal524R mutant CHIKV strains were produced from electroporation of *in vitro*-transcribed RNA of each infectious clone (mMessage mMachine SP6 transcription kit; Life Technologies, Inc.) into baby hamster kidney (BHK-21) cells (ATCC CCL-10). African green monkey Vero cells (ATCC CCL-81), human lung fibroblast MRC-5 cells (ATCC CCL-171), and *A. albopictus* C6/36 cells (ATCC CRL-1660) were used to obtain growth curves. Vero cells were used to perform all plaque assays.

### Mice.

All mice used in this study were C57BL/6J mice purchased from the Jackson Laboratory and bred in house. Twenty-four-day-old mice were inoculated subcutaneously in the left rear footpad with 100 PFU of CHIKV in phosphate-buffered saline (PBS) supplemented with 1% fetal bovine serum (FBS). Mock-infected mice were inoculated with diluent alone. Mice were weighed daily and monitored for clinical signs of disease. Footpad swelling was measured using calipers. Mouse sacrifice was performed using isoflurane (Attane; Minrad, Inc.) overdose. Animal husbandry and experiments were conducted under biosafety level 3 conditions and followed approved guidelines set forth by the University of North Carolina at Chapel Hill Institutional Animal Care and Use Committee.

### Sequencing of a CHIKV isolate.

The Caribbean isolate of CHIKV used in this study was amplified once on C6/36 cells. At 30 h postinfection, supernatants were collected and virus was purified on a 20% sucrose gradient by ultracentrifugation at 24,000 × *g* at 4°C overnight. Purified virus was resuspended in 1× PBS. RNA was isolated from purified virus preparations by TRIzol extraction (Ambion). Random priming was performed by annealing random primer 9 (New England Biolabs) to total RNA samples. cDNA was reverse transcribed using SuperScript II (Thermo, Fisher Scientific). Double-stranded DNA (dsDNA) was then produced by use of NEBNext mRNA second-strand synthesis module (New England Biolabs), and dsDNA was purified using a PureLink PCR microkit (Thermo, Fisher Scientific) according to standard procedures. Libraries were created using a Nextera XT DNA library preparation kit and sequenced on a MiSeq desktop sequencer using MiSeq reagent kit v2 (300 cycles) (Illumina). Coverage of the site analyzed included 35,377 reads.

### Growth curves.

Vero and C6/36 cells were infected in complete medium in triplicate at the indicated multiplicities of infection at 37°C or 28°C, respectively. At 1 h postinfection, inoculum was removed and cells were rinsed three times with PBS. Medium was replenished, and cells were incubated at 37°C or 28°C. At the times postinfection indicated in the figures, the supernatant was collected and stored at −80°C and medium was replenished to maintain a total volume of 1 ml per well.

### Virus titers.

The titers of all virus stocks, cellular supernatants, and tissue homogenates were determined by standard plaque assay on Vero cells. At the times postinfection indicated in the figures, mice were sacrificed by isoflurane overdose and perfused via intracardial injection with 1× PBS. Tissues designated for titration were harvested in 1× PBS and homogenized. All samples were stored at −80°C until the time of plaque assay.

### Sequencing of the Opal524R mutation in infected tissues.

Mice were sacrificed at 24 h postinfection, and the inoculated foot was removed. RNA was extracted from tissues using TRIzol (Ambion). Tissues were dissociated by homogenization prior to RNA extraction. Superscript III reverse transcriptase (Invitrogen) was used to generate cDNA by random priming from a total of 10 μg RNA from each sample. PCR was performed using Phusion polymerase (New England Biolabs) with primers flanking the opal stop codon to generate short amplicons (1 PCR per mouse; 5 mice for wild-type infections and 7 mice for Opal524R infections) that were analyzed by standard Sanger sequencing.

### Detection of nsP4.

A replicon plasmid that contains the pMH56.2 backbone encoding GFP in place of the structural genes was used. The Opal524R mutation was introduced into the replicon by restriction digestion of the Opal524R infectious clone and ligation into the replicon plasmid. A 3× FLAG tag was cloned at the C terminus of nsP4 in a two-step fusion PCR using the following primer sets: 5′CGGACACCGGTCCAGGTCATTTAC (forward), 5′CTACTTGTCATCGTCATCCTTGTAATCGATGTCATGATCTTTATAATCACCGTCAGGTCTTTGTAGTCTTTAGGACCGCCGTACAAAGTTATG (reverse 1), and 5′CACCATGGCGCGCCTGTAGCTGATTAGTGTTTAGATACTTGCTGTCGGCTTCTGCAAAATAGGTAGCTGTAGTGCGTACCTACTTGTCATCGTCATCCT (reverse 2). All constructs were sequenced across the opal stop codon and FLAG tag to confirm that no spurious mutations were introduced. RNA was *in vitro* transcribed from linearized plasmids (mMessage mMachine SP6 transcription kit; Life Technologies, Inc.) and purified by lithium chloride extraction. For each replicon, a total of 10 μg RNA was electroporated into BHK-21 cells.

### Western blotting.

Cells were rinsed in PBS, lysed in radioimmunoprecipitation assay (RIPA) buffer, and heated at 95°C for 5 min to ensure inactivation of infectious material. Lysates were clarified by centrifugation at 15,000 × *g* for 10 min, and protein was quantified by Bradford assay. Loading dye was added to equal quantities of protein from each sample, and these were resolved on SDS-PAGE gels. Protein was transferred onto polyvinylidene difluoride (PVDF) membranes, blocked for 1 h in 5 or 10% milk in Tris-buffered saline with Tween 20 (TBS-T), and probed overnight with the following antibodies: CHIKV nsP3 (mouse monoclonal, 1:500; produced in collaboration with Mary Ann Accavitti-Loper, University of Alabama Birmingham), CHIKV E2 (mouse monoclonal, 1:1,000; provided by Michael Diamond, Washington University), FLAG M2 (mouse monoclonal, 1:1,000; Sigma-Aldrich), and actin (goat monoclonal, 1:1,000; Santa Cruz Biotechnology). Secondary antibodies conjugated to horseradish peroxidase (HRP) were used against mouse (GE Healthcare) and goat (Sigma) primary antibodies.

### Densitometry analyses.

Bands were quantified for mature E2, nsP3, nsP34, or nsP4, where indicated, using ImageJ software (National Institutes of Health; version 1.48). Band densities were normalized to internal lane background densities. Band densities for individual viral proteins were then normalized to actin levels. Where indicated, band densities for duplicate lanes of an individual experiment were averaged.

### Histological analyses.

Mice were sacrificed and perfused intracardially with 4% paraformaldehyde. Tissues were embedded in paraffin, and 5-μm sections were prepared. Tissues were stained with hematoxylin and eosin. Slides were deidentified and scored for inflammation and musculoskeletal tissue damage as described previously (36). Slides were imaged using an Olympus BX43 model microscope with CellSens software.

### Immunohistochemistry.

Immunohistochemical analysis of nitrotyrosine levels has been described previously (36). Briefly, tissues were fixed in 4% paraformaldehyde and embedded in paraffin, and 5-μm sections were prepared. Slides were deparaffinized with xylene, blocked in 10% donkey serum (Jackson ImmunoResearch), and stained with antibody for nitrotyrosine (Millipore). Biotinylated secondary antibodies were detected with streptavidin–DyLight-594 conjugate, and slides were mounted with ProLong antifade reagent gold (Invitrogen). Slides were imaged with an Olympus BX60 fluorescence microscope; images were collected with the iVision v.4.0.0 software (BioVision Technologies).

### Flow cytometry.

Mice were sacrificed and perfused with 1× PBS, and the ipsilateral foot was harvested in digestion medium (complete RPMI medium supplemented with collagenase A and DNase). Spleen was harvested from mock-infected mice in 1× PBS and used for control stains. All tissues were minced, and feet were further dissociated by shaking them for 1.5 to 2 h at 37°C. Tissues were then rinsed, lysed in ACK (ammonium chloride potassium) buffer, rinsed again, and plated in HFA (Hanks’ balanced salt solution [HBSS] supplemented with 1% FBS and 1% sodium azide). Tissues were stained for 30 min at 4°C with the following antibodies: fluorescein isothiocyanate (FITC)-anti F4/80 (EBioscience), phycoerythrin (PE)-anti CD115 (EBioscience), PETR (Texas Red with R-phycoerythrin)-anti CD11c (Invitrogen), peridinin chlorophyll protein (PerCP)-Cy5.5-anti Ly6C (BioLegend), PE-Cy7-anti LCA (EBioscience), eF450-anti CD11b (EBioscience), allophycocyanin (APC)-anti GR-1 (BioLegend), APCeF780-anti major histocompatibility complex class IIc (MHC-IIc) (EBioscience), FITC-anti CD3e (EBioscience), PE-anti NK1.1 (EBioscience), PETR-anti CD4 (Invitrogen), PerCP-anti CD8 (BD), PE-Cy7-anti LCA (EBioscience), and APC-anti B220 (EBioscience). Flow cytometry was performed on a Beckman Coulter, Inc., CyAn advanced digital processor (ADP) and analyzed using Summit software. Neutrophils were classified as either LCA^+^ CD11c^+^ or LCA^+^ CD11c^−^ Gr1^+^. Monocytes were classified as LCA^+^ CD11c^−^ CD11b^+^. T cells were classified as LCA^+^ CD3e^+^ and either CD4^+^ or CD8^+^. B cells were classified as LCA^+^ CD3e^−^ B220^+^. NK cells were classified as LCA^+^ CD3e^−^ NK1.1^+^.

### Quantitative real-time PCR.

Mice were sacrificed and perfused intracardially with 1× PBS. RNA was extracted from cells and tissues using TRIzol (Ambion). Tissues were dissociated by homogenization prior to RNA extraction. Superscript III reverse transcriptase (Invitrogen) was used to generate cDNA by random priming from a total of 10 μg RNA from each sample. Real-time PCR was performed using ABI Prism 7300 sequence detection system software (v1.4.0) and TaqMan Gene Expression master mix (Applied Biosystems). For absolute quantification of viral RNA, CHIKV sequence-specific primer probes were used as described previously (29). A standard curve was run in tandem using CHIKV plasmid. For quantification of host transcripts, relative quantification was performed using the ΔΔ*C*_*T*_ method (where *C*_*T*_ is threshold cycle). For all PCR targets, quantification was normalized to the amount of 18S rRNA present in the sample. Absolute quantification of 18S was performed using a standard curve.

### Cytokine and chemokine protein quantification.

Protein levels of cytokines/chemokines in clarified tissue homogenates were evaluated using a multiplex bead array assay. All the antibodies and cytokine standards were purchased as antibody pairs from R&D Systems or PeproTech. Individual magnetic bead regions (Luminex Corp.) were coupled to cytokine-specific capture antibodies according to the manufacturer’s recommendations. Conjugated beads were washed and kept at 4°C until use. Biotinylated polyclonal antibodies were used at twice the concentrations recommended for a classical enzyme-linked immunosorbent assay (ELISA) according to the manufacturer. All assay procedures were performed in assay buffer containing PBS supplemented with 1% normal mouse serum (Invitrogen), 1% normal goat serum (Invitrogen), and 20 mM Tris-HCl (pH 7.4). The assays were run using 1,500 beads per each set of cytokines measured per well in a total volume of 50 µl. A total of 50 μl of each sample was added to the well and incubated overnight at 4°C. After being washed, the beads were resuspended in 50 μl of assay buffer containing biotinylated polyclonal antibodies against the measured cytokines for 1 h at room temperature, washed again, and then resuspended in 50 μl of assay buffer containing 1 μg/ml solution of streptavidin-PE (Invitrogen). The plates were read on a Luminex MAGPIX platform. For each bead set, >50 beads were collected. The median fluorescence intensity of these beads was recorded and used for analysis with the Milliplex software using a 5P regression algorithm.

### Statistical analyses.

All data were analyzed in the statistical programing language R (v.3.0.2). Where indicated in the figures, analysis of variance (ANOVA) was used to determine statistical differences between data sets; Tukey’s honestly significant differences method was used *post hoc* to correct for multiple comparisons. Where applicable, * indicates a *P* value of less than 0.05 and ** indicates a *P* value of less than 0.01. Multiplex array data were analyzed by a nonparametric Mann-Whitney *U* test. The false-discovery rate was set to 0.1, and significance was determined using the Benjamini-Hochberg method for multiple-comparison corrections. Where applicable, * indicates significance under these criteria.
